# Nonlinear Trimodal Regression Analysis of Radiodensitometric Distributions to Quantify Sarcopenic and Sequelae Muscle Degeneration

**DOI:** 10.1155/2016/8932950

**Published:** 2016-12-27

**Authors:** K. J. Edmunds, Í. Árnadóttir, M. K. Gíslason, U. Carraro, P. Gargiulo

**Affiliations:** ^1^Institute for Biomedical and Neural Engineering, Reykjavík University, Menntavegur 1, 101 Reykjavík, Iceland; ^2^IRCCS Fondazione Ospedale San Camillo, Via Alberoni 70, Lido, 30126 Venezia, Italy; ^3^Department of Rehabilitation, Landspítali, Hringbraut, 101 Reykjavík, Iceland

## Abstract

Muscle degeneration has been consistently identified as an independent risk factor for high mortality in both aging populations and individuals suffering from neuromuscular pathology or injury. While there is much extant literature on its quantification and correlation to comorbidities, a quantitative gold standard for analyses in this regard remains undefined. Herein, we hypothesize that rigorously quantifying entire radiodensitometric distributions elicits more muscle quality information than average values reported in extant methods. This study reports the development and utility of a nonlinear trimodal regression analysis method utilized on radiodensitometric distributions of upper leg muscles from CT scans of a healthy young adult, a healthy elderly subject, and a spinal cord injury patient. The method was then employed with a THA cohort to assess pre- and postsurgical differences in their healthy and operative legs. Results from the initial representative models elicited high degrees of correlation to HU distributions, and regression parameters highlighted physiologically evident differences between subjects. Furthermore, results from the THA cohort echoed physiological justification and indicated significant improvements in muscle quality in both legs following surgery. Altogether, these results highlight the utility of novel parameters from entire HU distributions that could provide insight into the optimal quantification of muscle degeneration.

## 1. Introduction

Muscle degeneration, characterized by the progressive loss of muscle mass, strength, and function, has been consistently identified as an independent risk factor for high mortality in both aging populations and individuals suffering from neuromuscular pathology or injury [1–7]. When associated with aging, this phenomenon is defined as sarcopenia, and while its prevalence has been readily linked with profound decreases in both physical activity and vitality, a precise, quantitative method for defining its diagnosis and etiology remains unknown [8–10]. However, despite the absence of a universally accepted definition, extant clinical literature commonly acknowledges the association of sarcopenia with the loss of both skeletal muscle structure and function, and many mechanisms have been implicated to govern these changes [11–20]. In general, the noncontractile tissue infiltration typically associated with muscle degeneration, in accordance with a loss of muscle mass, confers an increased risk for frailty, disability, reduced mobility, and eventual hospitalization [21–24]. However, most importantly, sarcopenic muscle degeneration has been directly correlated to eventual mortality in middle-aged and elderly adults [25, 26]. With a suggested prevalence of over 50% in individuals aged over 80 years, it is clear that identifying a normative clinical definition for sarcopenia is of considerable importance in an increasingly aging world [7].

The potential mechanisms that govern muscle degeneration in sarcopenia have likewise been identified within the context of neuromuscular pathology or injury. Indeed, the dramatic deleterious changes in muscle composition and function exhibited in these patients have been implicated as accelerated analogues to the changes evidenced in sarcopenia. This notion is especially evident in patients with spinal cord injury (SCI), as paralysis from lower motor neuron denervation drastically reduces skeletal muscle mass and increases local muscle adiposity and fibrosis [27]. In addition to SCI, skeletal muscle degeneration due to illness, known as cachexia, has been analogously associated with increases in relative muscle adiposity, which has likewise been correlated to increased rates of cachectic patient mortality and morbidity [28–30]. However, the optimum metric for assessing these changes in skeletal muscle quality remains debated.

Identifying a quantitative gold standard for myological assessment would allow for the generalizability of translational myology studies to clinical practice, thereby supporting the indication of compensatory targets for clinical intervention. Indeed, there is much extant literature regarding the investigation of muscle quality and implicating its use as a comorbidity index [13, 31–40], but these studies generally share few commonalities between quantitative assessment methods. However, one metric that remains ubiquitous is the use of average radiodensitometric HU values from entire cross sections or volumes to describe the muscle quality. While this value might indeed characterize general shifts in adiposity, averaging image matrices in this regard has the likelihood of eliminating a vast amount of other distribution characteristics that could elucidate other subtle differences in the muscle.

As of yet, the use of entire radiodensitometric distribution to assess muscle quality remains unreported. The increasing prevalence of sarcopenic and cachexic muscle degeneration necessitates the establishment of a gold standard for quantitative myological assessment. This was the prime motive for this study, which herein reports the development of a novel nonlinear trimodal regression analysis methodology utilized with radiodensitometric distributions from CT scans of defined upper leg volumes of a healthy young adult, a healthy elderly subject, and an SCI patient with complete lower motor neuron denervation. This method is further tested with a cohort of total hip arthroplasty (THA) patients, whose operative and healthy legs were assessed both before and one year following surgery. Results from these assessments highlight the utility of utilizing entire HU attenuation value distributions and identify novel parameters that could provide further insight into how muscle degeneration can be optimally quantified.

## 2. Material and Methods

### 2.1. Subject Details and Recruitment

To first ascertain potential differences in muscle degeneration pathways, as evidenced by subtle changes in HU distributions, three subjects were utilized in the first part of this study. The first of these subjects was a healthy, 35-year-old adult male subject, and the second was a healthy 68-year-old elderly male. Both subjects' CT scans were obtained as part of a general volunteer dataset for research in our facility. The third subject was a 52-year-old male who had suffered a right pelvic mass infiltration of the sciatic nerve, which had to be partially sacrificed during surgery. Both skin sensation and voluntary anterior-external leg movement were rescued following surgery, but despite the progressive reinnervation of the thigh and posterior leg muscles, complete denervation of the* tibialis anterior* and severe, partial denervation of the* glutei* and posterior muscles of the thigh was confirmed one year after surgery. CT images at this time were obtained via academic collaboration with the RISE2-Italy project for the purposes of this study [41–43]. To further support the utility of the reported method, CT scans from healthy and operative legs of 15 primary THA patients were utilized. Patient data was obtained as part of our ongoing collaborative database with the Icelandic National Hospital (Landspitali, Reykjavik).

### 2.2. CT Acquisition and Soft Tissue Voxel Segmentation

**Figure 1:**
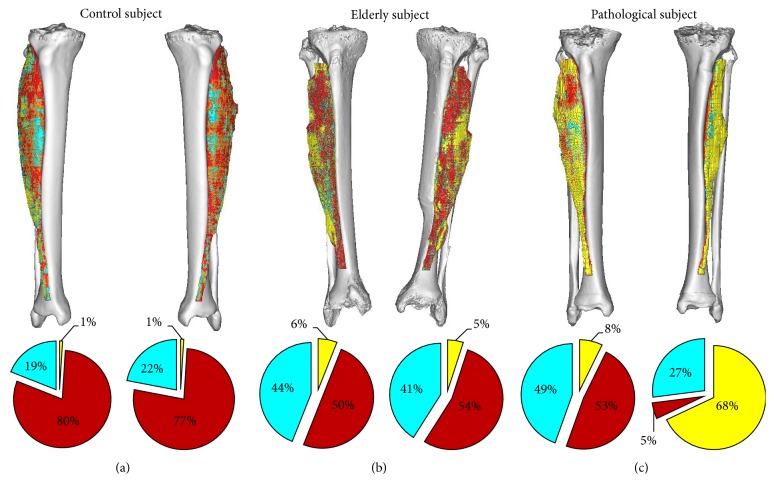
Segmented soft tissues and compositions within the* tibialis anterior* from (a) the healthy control subject, (b) the elderly subject, and (c) the pathological subject. Three tissue types of distinct radiodensitometric domains were utilized for the purposes of this study as follows: [−200 to −10], [−9 to 40], and [41 to 200] HU representing, respectively, fat (yellow), loose connective tissue and atrophic muscle (cyan), and normal muscle (red). Note that the fat voxel elements of the right (healthy) muscle of this patient were almost entirely superficial (visible on the surface of the segmentation model).

All participants in the project were scanned with a 64 Philips Brilliance spiral-CT machine. The scanning region extended from the iliac crest to the middle of the femur (Figure 1). The image protocol included slice thicknesses of 1 mm, with slice increments of 0.5 mm, and the tube intensity was set to 120 keV. In order to assemble 3D models of each patient's leg for soft tissue voxel segmentation, each patient's CT scan was imported into MIMICS Software (Materialise, Leuven, Belgium, available from http://biomedical.materialise.com/mimics). Tissue compositions within each leg volume were quantified by transforming CT numbers into HU values as previously reported [42]. These voxels were binned within the segmented volume into three HU intervals, which is evidenced in Figure 1 as follows: [−200 to −10], [−9 to 40], and [41 to 200] HU representing, respectively, fat (yellow), loose connective tissue and atrophic muscle (cyan), and normal muscle (red).

### 2.3. Voxel Distribution Binning

For each subject, HU distributions were derived from summing and transforming each voxel's CT number value according to the following linear transformation expression, defined by discretization of distributions into 128 CT bins from the total range [−200 to 200] as performed in literature [34, 42–45]:

(1) $$\begin{array}{c} HU=CT\times 3.125-200. \end{array}$$

Each resultant histogram was then exported for regression analyses. It should be noted that, for the purposes of comparing pathological muscle degeneration to sarcopenic degeneration, only the radiodensitometric distributions from subjects' left legs were utilized in the first part of this study. However, both the healthy and operative legs were utilized for the analysis of THA patients.

### 2.4. Statistical Analyses

Results from the THA cohort analyses were assessed for statistical significance by two-tailed heteroscedastic student's *t*-tests. Differences were considered statistically significant for *p* < 0.05.

### 2.5. The Method: Nonlinear Trimodal Regression Analysis

The method utilized to computationally define each HU distribution was a modified methodology for nonlinear regression analysis. First, the general equation for each distribution was defined as a quasi-probability density function by summing two skewed and one standard (*α* = 0) Gaussian probability density function (*φ*):  

(2) ∑i=13φx,μi,σi,αi=∑i=13Niσi2πe−x−μi2/2σi2erfc⁡αix−μiσi2,

where *N* is the amplitude, *μ* is the location, *σ* is the width, and *α* is the skewness of each distribution, all of which are iteratively evaluated at each CT bin, which is herein defined as the dependent variable, *x*. This definition is resultant from the hypothesis that each HU distribution is trimodal, in which they consist of three separate tissue types whose linear attenuation coefficients occupy distinct HU domains, namely, fat [−200 to −10 HU], loose connective tissue and atrophic muscle [−9 to 40], and normal muscle [41 to 200]. Additionally, we hypothesized that the inwardly sloping asymmetries within the fat and muscle peaks could be described by skewness (defined by the error function component, erfc) of their probability density functions, whereas the central connective tissue distribution was assumed to be a normal, nonskewed Gaussian distribution. Utilizing this definition, a theoretical curve was generated by employing an iterative generalized reduced gradient algorithm via minimization of the sum of standard errors at each CT bin value, *x*, thereby generating an 11-parameter matrix of probability density function variables. This algorithm iterates each function variable according to the computed variance of each step, and the selection of new trial values is guided by computing the rates of change of this variance as new inputs are generated. The minimization of the sum of standard errors at each point, and thereby the maximization of the coefficient of determination, *R*^2^, was computed according to standard definitions [46].

**Figure 2:**
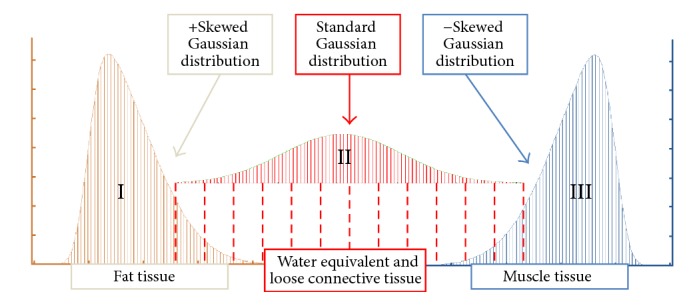
Diagram depicting the three components of the trimodal radiodensitometric distribution utilized in this study. This figure illustrates the location and skewness of each probability density function, with tissue types as previously defined.

An illustration of the results of this concept is shown in Figure 2, where each of the three tissue types and their respective probability density functions have been depicted.

## 3. Results and Discussion

### 3.1. Initial Case Studies: A Comparison of Degeneration Pathways

**Figure 3:**
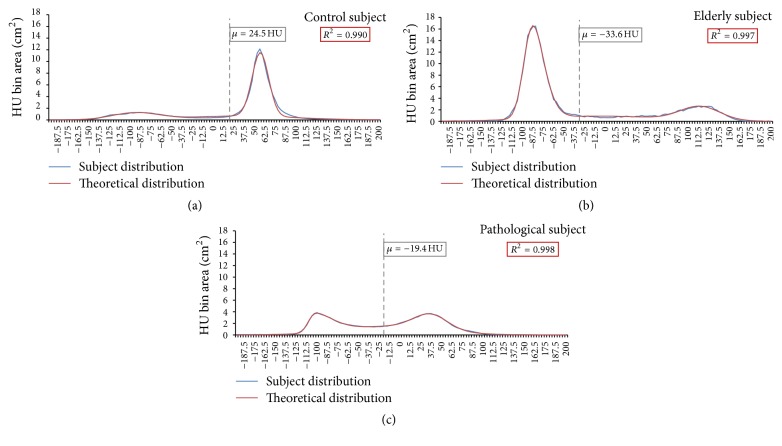
Radiodensitometric distributions showing their respective nonlinear regression curves and average HU values. (a) The control subject's curve showed a large muscle peak at around 55 HU, which directly contrasted with (b) the elderly subject's distribution. (c) The pathological subject's distribution was much lower in total pixel count (due to lower overall mass within the leg volume) and elicited fat and muscle peaks that are similar in amplitude with a large connective tissue regime between them.

As is evident from the results displayed in Figure 3, there are significant qualitative differences between the shapes of the HU distributions of the healthy, elderly, and pathological subjects. The curve of the healthy subject exhibits a definitively high-amplitude muscle peak and a comparatively blunted fat peak, whereas the fat and muscle components in the elderly subject's curve are decidedly the opposite in appearance. Contrastingly, the pathological subject elicited a distribution with heavily skewed fat and muscle peaks which were likewise closer together and shifted towards negative HU values.

**Figure 4:**
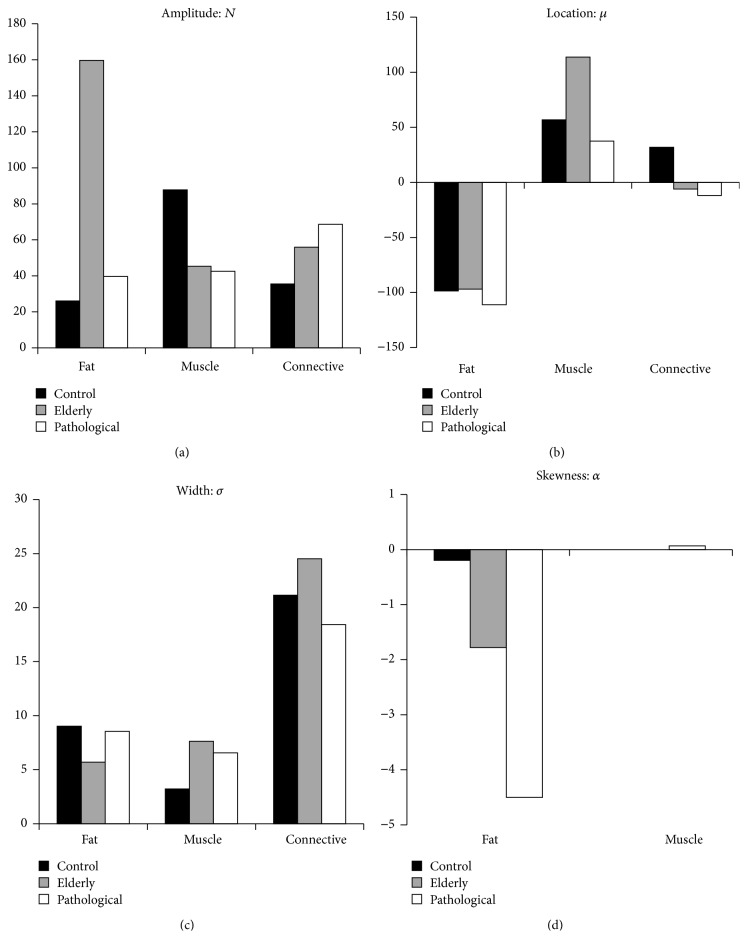
Results from the three representative subjects' nonlinear trimodal regression analyses. (a) The amplitude parameter,* N*; (b) the location parameter, *μ*; (c) the width parameter, *σ*; (d) the skewness parameter, *α*.

When compared according to the typical metric of average HU value, it is evident that the healthy subject's average HU value was significantly shifted towards the muscle peak in the distribution. However, the average HU values of the elderly and pathological subjects were nearly indistinguishable from one another. To better explore the clearly obtuse differences in their distributions, each regression analysis parameter was compiled and compared for the three subjects. The qualitatively distinguishable differences between HU distributions are further exemplified by the results from regression analyses and are compiled in Figure 4.

As is evident in Figure 4, each of the distribution parameters confers its own distinct differences and relationships between subjects. The amplitude parameter, *N*, elicits particularly intriguing results when accounting singular tissue types; the elderly subject's fat amplitude is at least fourfold larger than those of the other subjects, and the control subject's muscle amplitude is largest by at least twofold. However, the connective tissue amplitude is highest in the pathological subject; intriguingly, these values increase nearly linearly between subjects, with the lowest connective tissue amplitude in the control subject. These data are qualitatively apparent in the muscle and fat tissue peaks but somewhat less obvious in the central connective tissue peak. It is important to recall that our definition for the connective tissue distribution accounts for water-equivalent and loose-fibrous tissues that are always part of healthy leg volumes, but degraded, unhealthy muscle with aforementioned significant fatty infiltration would likewise populate this central HU attenuation region. This notion is described very well by the connective tissue amplifications progressively increasing from the control subject to the elderly and pathological subjects.

An analogous linearity is apparent when observing the fat tissue skewness, which is almost zero in the control subject and most extreme (highly negative) in the pathological subject. Interestingly, muscle skewness was zero in the elderly and control subjects and nonzero but very small (0.07) in the pathological subject. These data suggest that the fat peak's positive asymmetry could likewise be due to the progressive infiltration of fatty tissue into the much higher HU value muscle tissue. However, as this skewness relationship is not commensurate in the muscle peak, it remains unclear whether skewness as a variable can completely describe muscle degeneration.

The location parameter is almost identical between subjects' fat distributions, but the muscle peak location was singularly high in the elderly subject. Likewise, the control subject had a singularly positive connective tissue HU location, whereas the other subjects' values were negative. While less significant, perhaps, these results are still intriguing, showing that the central connective tissue regime of the HU distribution shifted towards more negative, “fatter” HU values in the elderly and pathological subjects. However, the fat peaks remained unshifted, and, unexpectedly, the muscle peak was higher in HU value in the elderly subject and nearly identical in the control and pathological subjects.

Finally, the width parameter exhibited noticeable differences between subjects. The control subject had the widest fat distribution, but the control muscle width was at least twofold lower than the other subjects. Likewise, while the elderly and pathological subjects had similar fat and muscle widths, the elderly subject elicited a comparatively much higher connective tissue distribution width. The physiological interpretation of width as a parameter is somewhat obscure, but one could argue that a sharply defined muscle and/or fat peak might suggest a comparative reduction in muscle degeneration. This notion is supported by the control subject's muscle peak being remarkably lower in width compared to the other subjects', but this is unsupported by the fat peak results.

### 3.2. Assessing Changes in Muscle following Total Hip Arthroplasty

As previously mentioned, the potential utility of the reported method was further tested with a cohort of 15 THA patients to assess changes in their upper leg muscle following surgery. To do this, HU distributions from each patient were acquired from both presurgical and one-year postoperative CT scans. Each distribution parameter was analyzed for both healthy and operative legs, and differences were assessed for statistical significance.

**Figure 5:**
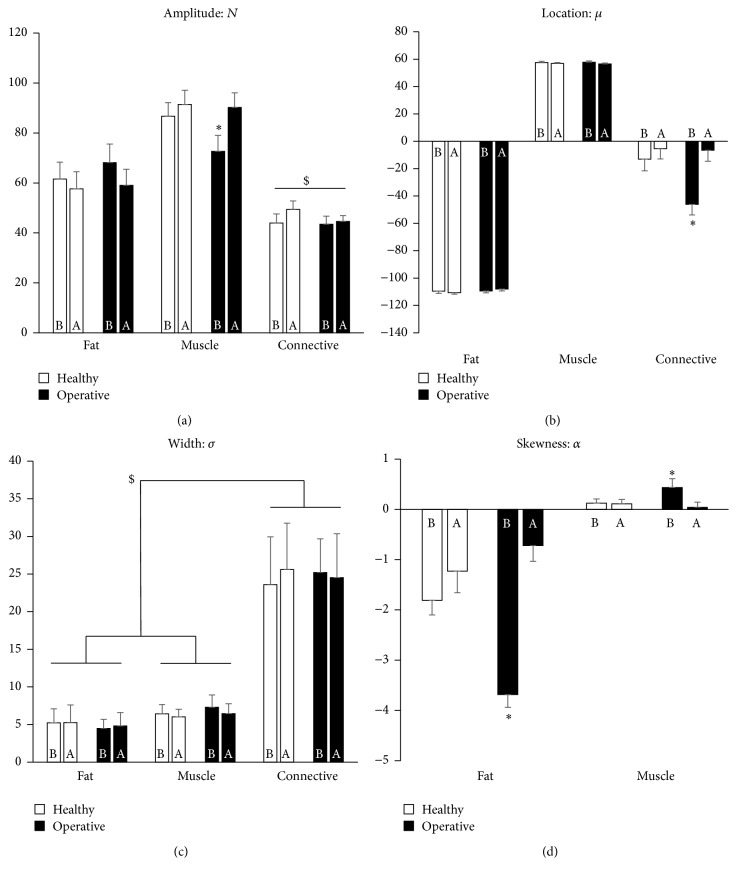
Results from the nonlinear trimodal regression analyses of the *n* = 15 THA cohort. (a) The amplitude parameter,* N*; (b) the location parameter, *μ*; (c) the width parameter, *σ*; (d) the skewness parameter, *α*. Note that $ and *∗* denote *p* < 0.05, and the results are presented before (b) and one year after (a) surgery.

As is evident in Figure 5, the results from our THA cohort analyses further support many aforementioned relationships between regression parameters and the degree of muscle degeneration, if one operates under the physiological assumption that patient operative legs would naturally be less utilized than their healthy legs. In general, fat amplitudes decreased while muscle amplitudes increased one year after surgery. However, it was only in the operative legs that a significant increase in muscle amplitude was observed. Connective tissue amplitudes were all significantly lower than fat and muscle.

Regarding the location parameter, there were minimal shifts evident in muscle and fat peaks, but commensurate with previous observation, there were notable increases in connective tissue location values in both the healthy and operative legs one year after surgery. This suggests the notion that connective tissue distributions may shift towards healthy muscle following one year of corrective ambulation and normative use. Indeed, once again, this was most evident and singularly significant in the operative leg.

The width parameter elicited no significant or meaningful changes in either leg, but in accordance with previous observation, the connective tissue peak widths were significantly larger than either the muscle or fat peaks, which were both nearly identical in magnitude in both legs. While not apparently useful, it still remains intriguing that each distribution parameter seems to have its own sensitivity with respect to the population.

Finally, the skewness parameter decreased in magnitude in both the healthy and operative fat peaks but remained relatively constant and small (less than one) in the muscle peak, with one exception: the preoperative muscle peak was significantly higher than each of the others. We previously saw that the pathological subject had a much more extreme (more negative) fat skewness than the other two initial subjects, suggesting the infiltration and/or buildup of intramuscular fat in his degenerating muscle. This was once again similarly evident here, as the operative legs of the THA cohort elicited significantly more extreme, negative skewness than the rest of the fat peak values.

Altogether, these results indicate significant improvement in muscle quality in both legs following surgery, a notion which is most evident in patient operative legs. These data further support the notion that each HU distribution parameter may have a particular range of specificity when it comes to muscle assessment, thereby suggesting the method's utility as a straightforward indicator for muscle degeneration.

### 3.3. Exploring the Partial Volume Effect

One of the more commonly discussed topics regarding tissue segmentation from medical images is that of the Partial Volume Effect (PVE). This phenomenon may be defined as the loss of fidelity in small regions or morphologies from limitations in spatial resolution of a particular imaging modality, and PVE is of particular relevance in positron emission tomography (PET) and dissemination of intracranial tissues using magnetic resonance imaging (MRI) [47]. In regard to our study here, one might argue that it may be necessary to initially correct pixels on the boundary of muscle groups and subcutaneous fat, due to the PVE being highest in these pixels. While this may indeed allow for a better fat to muscle segmentation fidelity, it may be argued that the degradation of myofibers would readily dictate the prevalence of PVE within our CT images. The correction of boundary pixels would therefore correct the very pixels we wish to consider in our distributions, as it is clear that their presence could be utilized as a supportive metric for assessing muscle degeneration.

**Figure 6:**
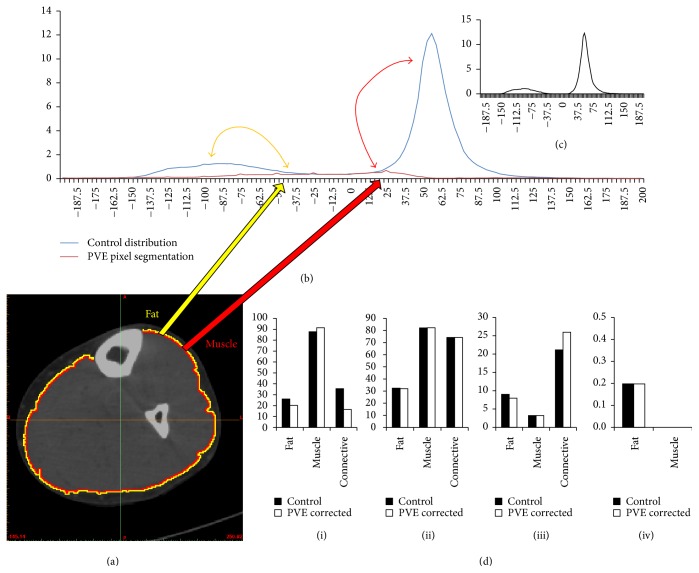
Results from Partial Volume Effect pixel removal using the healthy control subject. (a) Transverse CT cross section of the control subject's leg with two pixel boundary layers segmented and corrected. (b) Distributions illustrating the control subject HU distribution and the segmented PVE pixel layer distribution. (c) Resultant distribution showing an almost nonexistent connective tissue central peak. (d) Results from the corrected distributions with each parameter: (i) *N*; (ii) *μ*; (iii) *σ*; (iv) *α*.

To test this notion, we took the control subject's HU CT scan and segmented a two-pixel wide boundary layer between fat and muscle tissues. These pixels were then subtracted from the distribution at their given HU values and redistributed to either the fat or muscle mean HU value based on their respective proximities to either tissue. The results from this analysis are shown in Figure 6. As is evident from these results, the subtraction of these pixels resulted in a great reduction in the central connective tissue distribution and elicited minimal changes in regression analysis parameter values. However, the significant reduction in the water-equivalent and loose connective tissue peak highlights the possibility for PVE correction to remove useful data, especially considering that degenerated muscle and infiltrative adipose tissue would most likely exist in this region.

It is likewise useful to assess these subjects according to their average HU value, which is the oft-cited method for CT analysis, as previously mentioned. As is evident in Figure 3, this analysis gives no clear distinction between the elderly and pathological patients, but the healthy subject's average HU value is much higher than the others. These results suggest that the use of the average HU value cannot distinguish between elderly and pathological distributions, despite clear evidence that there are significant differences in how their muscle degeneration is evidenced by our radiodensitometric curve parameters.

## 4. Conclusions

While there is much extant literature reporting the use of average HU values to investigate muscle quality and its utility as a comorbidity index, no studies have yet to utilize the entire radiodensitometric distribution. The increasing prevalence of sarcopenic and cachexic muscle degeneration necessitates the establishment of a robust quantitative myological assessment methodology. Herein, we hypothesize that rigorously quantifying entire HU distributions can elicit much more information regarding muscle quality than extant methods. This study reports the development and use of this method, wherein we assess upper leg muscle quality utilizing nonlinear trimodal regression analysis with radiodensitometric distributions from computed tomography (CT) scans of a healthy young adult, a healthy elderly subject, and a spinal cord injury patient exhibiting complete lower motor neuron denervation. We show that physiological justification for these initial results is yet again evidenced by the use of a cohort of total hip arthroplasty (THA) subjects. While the use of more subjects will be essential to reinforcing the physiological claims reported here, these results altogether highlight the potential utility of our method and the importance of utilizing entire HU attenuation value distributions. We have likewise identified a host of novel regression parameters from these analyses that could provide further insight into how muscle degeneration can be optimally quantified. These notions support the conclusion that our method may be a pivotal first step in the development of a new gold standard for the analysis of muscle.
